# Development and Validation of a Nomogram for Predicting Pathological Intussusceptions in Children Prior to Surgical Intervention

**DOI:** 10.3389/fped.2022.877358

**Published:** 2022-07-18

**Authors:** Xu Ting, Duan Xufei, Liu Jiangbin, Xu Weijue, Lv Zhibao, Ye Guogang

**Affiliations:** ^1^Department of General Surgery, School of Medicine, Shanghai Children’s Hospital, School of Medicine, Shanghai Jiao Tong University, Shanghai, China; ^2^Department of General Surgery, Tongji Medical College, Wuhan Children’s Hospital, Huazhong University of Science and Technology, Wuhan, China

**Keywords:** intussusception, pathological intussusception, nomogram, children, pathologic lead points

## Abstract

**Purpose:**

Establish and validate a nomogram to help predict the preoperative risk of a pathological intussusception.

**Methods:**

A primary cohort of patients who underwent surgery for an intussusception were enrolled from one center, while a validation cohort consisted of patients from another center. Multivariate logistic regression analysis was used to identify the variables to build the nomogram. A calibration curve accompanied by the Hosmer-Lemeshow test was used to assess the calibration of the nomogram. To quantify the discrimination of the nomogram, Harrell’s C-index was calculated. The performance of the validated nomogram was tested in the external validation cohort. The logistic regression formulae created during the analysis of the primary cohort was applied to all patients in the external validation cohort, and the total points for each patient were calculated.

**Results:**

The primary cohort consisted of 368 patients and the validation cohort included 74. The LASSO logistic algorithm identified three (recurrence episodes, mass size, and infection history) out of 11 potential clinical variables as significantly predictive of a pathologic intussusception. The C-index for the predictive nomogram was 0.922 (95% CI, 0.885–0.959) for the primary cohort and 0.886 (95% CI, 0.809–0.962) for the validation cohort. The decision curve showed that if the threshold probability of a patient in the validation cohort was > 7%, then the nomogram was more beneficial than either indiscriminately treating all or none of the patients.

**Conclusion:**

We developed a nomogram based on clinical risk factors that could be used to individually predict pathological intussusceptions in children prior to surgical intervention.

## Introduction

Intussusception is one of the most common causes of an acute abdomen in the pediatric emergency clinic and is the leading cause of acute bowel obstruction in infants and young children (1–3). In general, most intussusceptions can be categorized as primary or idiopathic, with thickened bowel wall lymphoid tissue (Peyer patches) acting as the lead point. Approximately 5–12% of intussusceptions are pathological, which usually present with different symptoms and are caused by conditions such as Meckel’s diverticula (4), intestinal polyps (5, 6), intestinal duplication (7), allergic purpura (8) and some rare digestive tract malignant tumors (9, 10).

Although diagnosing an intussusception is relatively easy, an expanded differential diagnosis in the setting of a pathological intussusception remains a challenge for most pediatricians, radiologists and pediatric surgeons. The initial management of an intussusception in most cases is non-surgical reduction *via* air or barium enema. Operative reduction is only needed when there is suspicion of bowel necrosis and perforation or non-surgical reduction fails. When a pathological intussusception is caused by pathologic lead points (PLPs), surgical treatment is ultimately required. Surgery should not be delayed in children in the setting of a difficult or doubtful pneumatic reduction, especially in older children. Timely diagnosis and treatment of intussusception avoids serious complications such as bowel necrosis, perforation, and death (11, 12).

Doctors usually order an ultrasound or CT scan when PLPs are suspected in patients with intussusceptions, but these have low sensitivity and specificity (13, 14). If a pathological intussusception could be diagnosed and treated earlier using advanced diagnostic capabilities, discomfort caused by repeated enemas would be avoided and prognosis would improve (15, 16). There exists a need to establish a more accurate, non-invasive and reusable diagnostic model for diagnosing pathological intussusceptions. Several models that have already been established are not perfect, and few focus specifically on the risk factors for a pathological intussusception. The aim of this study was to develop and validate a diagnostic nomogram based on the clinical and pathologic risk factors for individual pre-operative risk prediction.

## Patients and Methods

### Patients and Study Design

A retrospective study was conducted on a primary cohort of patients who underwent surgery for intussusception between January 2015 and December 2019 at the Department of General Surgery at Shanghai Children’s Hospital. A total of 368 patients with intussusceptions were enrolled in this study. These patients formed the primary cohort. Inclusion criteria were patients with intussusceptions that were treated with surgery after three failed air enema reductions. Neonatal intussusceptions, postoperative intussusceptions and patients with successful air enemas were excluded. Using the same inclusion and exclusion criteria, the external validation cohort consisted of 74 patients who underwent surgery for intussusceptions at Wuhan Children’s Hospital between January 2018 and December 2019.

Most of the cases in our study were diagnosed on abdominal ultrasound, the gold standard for intussusception diagnosis, while a few patients were diagnosed with computed tomography (CT) and air enemas because ultrasound was not available. Patients who were successfully treated *via* air enema reduction were discharged from the hospital. If the air enema reduction did not succeed after three attempts, patients were admitted for immediate surgery. Recurrent intussusceptions were defined as intussusceptions that recurred within 12 h of a successful air enema reduction. Multiple-recurrent intussusceptions were defined as cases that recurred at least twice after air enema reduction or surgery, with episodes defined as the number of times that the intussusception recurred after air enema reduction or surgery.

The ultimate diagnosis of a pathological intussusception was made by the pathologist based on post-operative histopathological findings. Clinical and pathological data were collected, including gender, age, weight, abdominal pain time, season, vomiting, episode times, mass length, bloody stools, and infection history. Mass length was defined as that measured on ultrasound or the largest length on axial CT cuts. Infection history included respiratory and digestive symptoms in the preceding week, such as coughing, rhinorrhea, and diarrhea. All information was obtained at the time of the first reduction by air enema or surgery. Each case was followed for 1 year from the time of the first intussusception.

### Development of the Nomogram

Multivariable logistic regression analysis began with the following clinical candidate predictors: gender, age, weight, abdominal pain time, season, vomiting, episode times, mass length, bloody stool, and the infection history. A backward step-wise regression was applied using the likelihood ratio test with Akaike’s information criterion as the stopping rule (17, 18). To provide an easy and personalized quantitative tool for predict the probability of a pathological intussusception we built the nomogram based on variable that were statistically significant in the multivariate logistic model in the primary cohort.

Apparent performance of the nomogram in the primary cohort calibration curve was plotted to assess the calibration of the nomogram and the Hosmer-Lemeshow test was performed (a significant test statistic implies that the model does not calibrate perfectly) (19). To quantify the discriminatory performance of the nomogram, Harrell’s C-index was measured. To correct overfitting bias, a corrected C-index was calculated using bootstrapping validation (1000 bootstrap resamples) in the primary cohort.

### Validation of the Nomogram

The performance of the validated nomogram was tested in the external validation cohort. The logistic regression formula formed in the primary cohort was applied to all of the patients in the external validation cohort, with total points calculated for each patient. Logistic regression in this cohort was then performed using total points as a factor. Finally, C-index and calibration curve were derived from the regression analysis.

### Clinical Use

The clinical usefulness of the nomogram was evaluated by quantifying its net benefits at different threshold probabilities in the validation cohort using decision curve analysis (20, 21). True positive (TPR) and false positive rates (FPR) were also calculated in the validation cohort at different threshold probabilities. The corrected number of positive patients identified with the nomogram and the number of actually positive patients were calculated using bootstrapping validation (1000 bootstrap resamples) in the validation cohort.

### Statistical Analysis

Statistical analysis was performed with R software (version 3.5.0, R Foundation for Statistical Computing), with *P*-values less than 0.05 considered statistically significant. Normally distributed data were expressed as mean ± *SD*, and non-normally distributed data were expressed as median (Q1, Q3). Categorical data were expressed as a number and percentage and compared using the Chi-square or Fisher’s Exact tests. Continuous data were compared using Student’s *t*-test or Wilcoxon’s rank test.

The most meaningful clinical characteristics in the prediction of a pathological intussusception were selected by least absolute shrinkage and selection operator (LASSO) analysis. Identification of independent clinical characteristics that were predictive of a pathological intussusception was performed using multivariate logistic regression analysis, permitting the creation of a predictive model. A nomogram containing those predictive factors was also assessed for its calibration, discrimination, and clinical usefulness.

## Results

### Clinical Characteristics

The incidence of a pathological intussusception was 19.3% (71/368) and 20.3% (15/74) in the primary and validation cohorts, respectively. There were no significant differences between the two cohorts in recurrent intussusceptions (*P* = 0.841) or clinical characteristics. The characteristics of all of the cases in the primary and validation cohorts are shown in Table 1. Age, weight, episode time, mass length and infection history were all positively correlated with a pathological intussusception in both the primary and validation cohorts (Table 2). The main types of PLPs were benign polyps (*n* = 25), Meckel’s diverticula (*n* = 21) and duplication cysts (*n* = 9) in the primary cohort. All PLP types are shown in Table 3.

**TABLE 1:** Demographics and clinicopathologic characteristics of children with intussusceptions.

Demographics or characteristic	Primary cohort *n* = 368	Validation cohort *n* = 74	*P*-value
Sex, *n* (%)			0.563
Male	251 (68.2)	53 (71.6)	
Female	117 (31.8)	21 (28.4)	
Age (months)			0.193
Median	11	12	
Inter-quartile range	7–34	8–36	
Weight (kg)			0.227
Median	11	12	
Inter-quartile range	8–15	8–16	
Pain time (h)			0.096
Median	8	9	
Inter-quartile range	6–11	7–12	
Season, *n* (%)			0.931
Spr/Aut	191 (51.9)	38 (51.4)	
Sum/Win	177 (48.1)	36 (48.6)	
Vomiting, *n* (%)			0.158
Yes	317 (86.1)	59 (79.7)	
No	51 (13.9)	15 (20.3)	
Times			0.123
Median	1	1	
Inter-quartile range	1–2	1–2	
Length of mass, *n* (%)			0.325
≥ 8 cm	94 (25.5)	23 (31.1)	
< 8 cm	274 (74.5)	51 (68.9)	
Bloody stool, *n* (%)			0.889
Yes	222 (60.3)	44 (59.5)	
No	146 (39.7)	30 (40.5)	
Infection, *n* (%)			0.113
Yes	249 (67.7)	43 (58.1)	
No	119 (32.3)	31 (41.9)	
Type, *n* (%)			0.841
Primary	297 (80.7)	59 (69.7)	
Pathological	71 (19.3)	15 (20.3)	

**TABLE 2:** Demographics and clinicopathologic characteristics of children in the primary and validation cohorts.

Demographics or Characteristic	Primary cohort			Validation cohort		
	Pri (297)	Pat (71)	*P*-value	Pri (59)	Pat (15)	*P-*value
Sex, *n* (%)			0.904			0.421
Male	203 (68.4)	48 (67.6)		41 (69.5)	12 (80.0)	
Female	94 (31.6)	23 (32.4)		18 (30.5)	3 (20.0)	
Age (months)			<0.001			0.001
Median	9	42		12	55	
Inter-quartile	7–21	12–75		8–23	12–84	
Weight (kg)			<0.001			0.002
Median	9	17		11	18	
Inter-quartile	8–13	11–22		8–13	11–24	
Pain time (h)			0.455			0.142
Median	8	9		9	10	
Inter-quartile	6–10	7–11		7–11	8–14	
Season, *n* (%)			0.309			0.453
Spr/Aut	158 (53.2)	33 (46.5)		29 (49.2)	9 (60.0)	
Sum/Win	139 (46.8)	38 (53.5)		30 (50.8)	6 (40.0)	
Vomiting, *n* (%)			0.657			0.357
Yes	257 (86.5)	60 (84.5)		48 (81.4)	11 (73.3)	
No	40 (13.5)	11 (15.5)		11 (18.6)	4 (26.7)	
Times			<0.001			<0.001
Median	1	3		1	3	
Inter-quartile	1–1	3–4		1–2	3–4	
Mass size, *n* (%)			<0.001			0.001
≥ 8 cm	41 (13.8)	53 (74.6)		13 (22.0)	10 (66.7)	
< 8 cm	256 (86.2)	18 (25.4)		46 (78.0)	5 (33.3)	
Bloody stool, *n* (%)			0.261			0.588
Yes	175 (58.9)	47 (69.0)		36 (61.0)	8 (53.3)	
No	122 (41.1)	24 (31.0)		23 (39.0)	7 (46.7)	
Infection, *n* (%)			<0.001			0.001
Yes	243 (81.8)	6 (8.5)		40 (67.8)	3 (20.0)	
No	54 (18.2)	65 (91.5)		19 (32.2)	12 (80.0)	

**TABLE 3:** Pathology of patients with intussusceptions in the primary cohort.

Pathology	No. of patients
Polyps	25
Meckel’s diverticulum	21
Intestinal duplications	9
Lymphomas	6
Adenomas	3
Allergic purpura	4
Heterotopic pancreas	2
Adenomyomas	1

### Independent Prognostic Factors in the Primary Cohort

**FIGURE 1:**
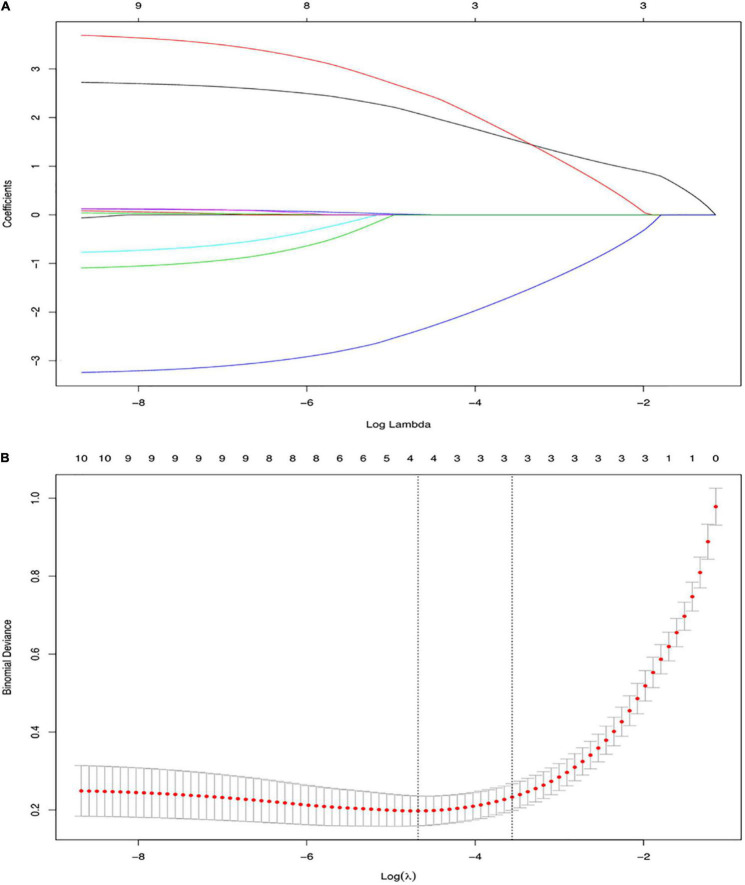
Variable selection using the least absolute shrinkage and selection operator (LASSO) binary logistic regression model. **(A)** LASSO coefficient profiles, displaying 11 variables. A coefficient profile plot was produced against the log (lambda) sequence. Each colored line represents the coefficient of an individual feature. **(B)** Tuning parameter (log lambda) selection in the LASSO model used tenfold cross-validation *via* minimum criteria. Vertical dotted lines were drawn at the selected λ values. LASSO, Least Absolute Shrinkage and Selection Operator.

Three out of the 11 clinical candidate predictors were used to develop the nomogram (Figure 1). The results of the univariate and multivariate analyses are listed in Table 4. Multivariate analysis demonstrated that episode time, mass length and infection history were independent risk factors for a pathological intussusception.

**TABLE 4:** Logistic analysis of the primary cohort.

Variable	Univariate analysis		Multivariate analysis	
	HR (95% CI)	*P*	HR (95% CI)	*P*
Sex	1.035 (0.595–1.801)	0.904	0.905 (0.199–4.124)	0.898
Age (months)	1.037 (1.026–1.047)	0.000	1.049 (0.898–1.225)	0.549
Weight (kg)	1.167 (1.115–1.221)	0.000	0.825 (0.385–1.769)	0.622
Pain time (h)	1.021 (0.967–1.077)	0.455	1.091 (0.895–1.330)	0.389
Season	1.309 (0.779–2.200)	0.309	2.205 (0.443–10.967)	0.334
Vomiting	1.178 (0.571–2.430)	0.657	0.758 (0.097–5.945)	0.792
Times	13.173 (7.588–22.868)	0.000	17.209 (5.712–51.846)	<0.001
Length of mass	18.385 (9.810–34.456)	0.000	40.891 (5.572–300.074)	<0.001
Bloody stool	1.365 (0.793–2.351)	0.261	2.568 (0.368–17.907)	0.341
Infection	0.021 (0.008–0.050)	0.000	0.041 (0.008–0.195)	<0.001

### Development of an Individualized Prediction Model

**FIGURE 2:**
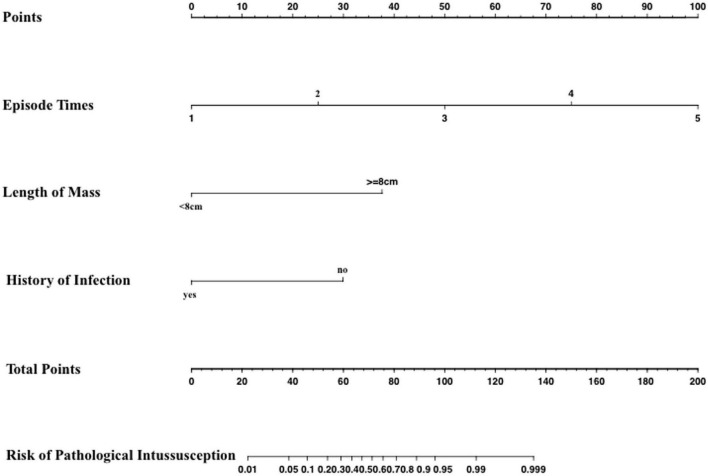
Nomograms for predicting the risk of a pathological intussusception.

A logistic regression analysis identified episode time, mass length, and infection history as independent predictors of a pathological intussusception (Table 2). A model that incorporated the above independent risk factors was developed and presented as the nomogram (Figure 2).

### Apparent Performance of the Nomogram in the Primary Cohort

The C-index for the prediction nomogram was 0.922 (95% CI, 0.885–0.959) in the primary cohort. The calibration curve demonstrated a good agreement between prediction and observation in the training cohort. The Hosmer–Lemeshow test found no statistical significance in the training cohort (*P* = 0.366), which suggested that there was no departure from a perfect fit.

### Validation of the Nomogram

Good calibration was observed for the probability of a pathological intussusception in the validation cohort using the nomogram. The Hosmer-Lemeshow test yielded a non-significant statistic (*P* = 0.591), And the C-index of the nomogram for the prediction of a pathological intussusception was 0.886 (95% CI, 0.809–0.962).

### Clinical Use

The decision curve analysis (DCA) for the nomogram was underwent by the R software. The decision curve showed that if the threshold probability of a patient in the validation cohort was > 7%, using the nomogram to predict a pathological intussusception added more benefit than either treat-all or treat-none strategies. Clinical impact curves were plotted and validated using the validation cohort. The curves demonstrated that a threshold probability of patients in the validation cohort of > 7% yielded good agreement between the number of positive patients labeled by the nomogram and the number of actually positive patients. TPR and FPR were satisfied by the nomogram in the prediction of a pathological intussusception at different threshold probabilities.

## Discussion

Here we developed and validated an effective preoperative nomogram that used clinicopathologic risk factors to predict a pathological intussusception in children. Episode time, mass length and the infection history were incorporated into the nomogram based on data from a relatively large database derived from two different hospitals. The novel nomogram is a non-invasive, easy-to-use preoperative tool to predict a pathological intussusception in children with intussusceptions that may reduce the need for air enemas or the need for additional radiological examinations. Though some of the cases underwent diagnostic CT scans in our study, we strongly recommend that a diagnostic air enema would have been a better choice due to higher risk of the radiation from the CT scan.

Our results suggest that episode time was an independent risk factor for pathological intussusceptions in children. Prior studies have reported a relationship between pathological intussusceptions and recurrent intussusceptions, as more than 3 recurrences makes a pathological intussusception highly likely (22, 23). Intussusception secondary to PLPs tended to recur frequently. The overall recurrence rate of all intussusception cases was extremely variable and depended on the series, ranging from 2 to 20% (23–26). Lin et al. (15) reported that 78.5% (51/65) of patients with intussusceptions secondary to PLPs recurred after their first episode. Among the 65 pathological intussusception patients in their work, 51 recurred after a successful reduction (21 with one recurrence, 14 with two, 10 with three, and 6 with more than three). Chen et al. (22) reported an overall recurrence rate of 20.0%, and the proportion of PLPs in recurrence group was higher than in the non-recurrence group (2.2% and 1.2%, *P* = 0.005). Univariate and multiple logistic regression analyses confirmed that PLPs were predictive of the intussusception recurrence in children (27), which was similar to our results. Furthermore, multiple authors (12, 28) emphasized that the presence of PLPs should always be considered in children with recurrent intussusceptions.

Zhang et al. (13) in their analysis of 37 cases of pathological intussusception reported that the length of the intussusception was significantly longer in pathological intussusceptions than in primary intussusceptions (*P* = 0.042), which could be explained by the existence of PLPs in pathological intussusceptions. Due to the presence of PLPs, the proximal retracted small bowel (intussusceptum) carries the mesentery into the distal bowel tract (sheath), so pathological intussusceptions would involve a longer length of bowel. We found in the present work that patients with pathological intussusceptions more commonly had intussusception lengths ≥ 8 cm compared with those with primary intussusceptions.

Multiple logistic regression analysis in our study confirmed that a history of infection, defined as symptoms and signs of fever, coughing, rhinorrhea, and diarrhea, was significantly less common in patients with pathological intussusceptions than primary intussusceptions. A history of infection was found to be an independent negative predictor of a pathological intussusception in children. Lee et al. (29) reported that a history of infection was significantly more common in non-recurrent (72/114, 63.2%) than recurrent (5/23, 21.7%) intussusceptions (*P* < 0.001). Park et al. (30) found that the incidence of pediatric intussusceptions decreased after the COVID-19 pandemic. That decreased incidence may be related to the reduced circulation of communicable diseases. However, the proportion of patients with more severe intussusceptions and PLPs rose (2/87 and 4/27). All of those findings are consistent with ours, fewer incidences of history of infection in pathological intussusception than in primary.

Our multivariate analysis showed that age was not a strong independent predictor of a pathological intussusception, which makes the exclusion of this variable a common strategy for model development. However, the rejection of potentially important predictors may be the result of nuances in the data set or confounding by other predictors (17). A non-significant statistical association with pathological intussusception therefore does not definitively imply that age is not predictive of this pathology. Zhang et al. (13) reported that the age of patients with pathological intussusceptions was older than those with primary intussusception. Lin et al. (15) further found that those patients older than 2 years of age, especially between 2 and 5 years of age, were more likely to have a pathological intussusception. Blakelock and Beasley (31) reported that 60% of PLPs occurred in children aged 5–14 years. Our nomogram that excluded age as a clinical risk factor demonstrated adequate discrimination in the primary cohort (C-index, 0.922), although it deteriorated in the validation cohort (C-index, 0.886). Given that the incidence of pathological intussusceptions was comparable between the two cohorts, even the reduced discrimination suggests that our nomogram is highly predictive and could be applied directly to validation cohorts.

Pathological intussusception with malignant tumors as their pathologic lead points are rare and mostly caused by Burkitt’s lymphoma (32, 33) in children. Only 6 out of 368 patients in our study had lymphoma. Their disease was limited to the area of the intussusception and was completely resected. However, prior works noted that some patients with stage III or IV disease could only undergo an incomplete surgical resection of their intussusception followed by additional chemotherapy, and had poor prognoses (33). Early diagnosis and treatment are therefore of great importance. We hope that the nomogram developed in the present study may allow these patients to have better outcomes.

Decision curve analysis was used to justify the clinical usefulness of our nomogram. Clinical impact curves showed that if the threshold probability of the is > 7%, there is good agreement between the number of positive patients by the nomogram and that of actually positive patients.

There are several limitations to this study. First, the nomogram was established based on data obtained from a single institution in China. Second, all data were collected retrospectively and bias therefore could not be avoided. Further prospective studies are needed to validate our model. Lastly, the universality of the nomogram for all intussusception patients remains unknown because some of the patients who had successful air enema reductions were excluded from this study. Further prospective analyses are required to validate our model in all patients with intussusceptions.

## Conclusion

In conclusion, we developed a nomogram based on clinical risk factors for a pathological intussusception. This nomogram can be conveniently used to facilitate pre-operative decision-making for children with intussusceptions.

## Data Availability Statement

The original contributions presented in this study are included in the article/supplementary material, further inquiries can be directed to the corresponding author.

## Ethics Statement

The studies involving human participants were reviewed and approved by the Ethics Committee of Shanghai Children’s Hospital (2018R055). Written informed consent to participate in this study was provided by the participants’ legal guardian.

## Author Contributions

YG and LZ: conception and design. XT and DX: administrative support and collection and assembly of data. XT, DX, and XW: provision of study materials. XT, YG, and LJ: data analysis and interpretation. All authors listed have made a substantial, direct, and intellectual contribution to the work, approved it for publication, and wrote the manuscript.

## Conflict of Interest

The authors declare that the research was conducted in the absence of any commercial or financial relationships that could be construed as a potential conflict of interest.

## Publisher’s Note

All claims expressed in this article are solely those of the authors and do not necessarily represent those of their affiliated organizations, or those of the publisher, the editors and the reviewers. Any product that may be evaluated in this article, or claim that may be made by its manufacturer, is not guaranteed or endorsed by the publisher.
